# The publication symmetry test: a simple editorial heuristic to combat publication bias

**Published:** 2017-07-31

**Authors:** Brian D. Earp, Dominic Wilkinson

**Affiliations:** ^1^Departments of Psychology and Philosophy, Yale University, New Haven, Connecticut, United States; ^2^Oxford Uehiro Centre for Practical Ethics, University of Oxford, Oxford, England, United Kingdom

Premier academic journals-that is, the journals in which many researchers must publish their work in order to maintain or advance their careers-have historically tended to reject papers reporting "negative" or null findings, including those derived from "failed" attempts to replicate prior results [1-3]. This tendency was likely due to three main factors. First, the limited space available for publishing articles when journals were printed exclusively on paper. Second, the prestige-related desire of "top" journals to publish new and exciting findings-i.e., "discoveries" (often taken to imply a demonstration that something "works," as opposed to "fails to work"). And third, the difficulty posed by negative findings in terms of how they should be interpreted: do they suggest that there is no effect of interest to be found, or rather that the experiment, whether in its design or execution, was simply inadequate to show the effect even though it is real [4-6]?

Journals are now mostly online, so page limits no longer provide a valid reason for failing to publish negative findings. There is still the matter of how to interpret such findings, but that, too, should not prevent publication of a well-designed and competently executed study [7,8]. Looking at the history of science, Stuart Firestein has shown that negative results have often been the wellspring of future discoveries and innovations [9]. Such results may have other benefits as well. Not only may they be valuable for researchers themselves-steering them away from wasting resources on likely dead ends-but also for our collective understanding of what we really know, for example, the true effectiveness of medical interventions [10-13]. This last consideration has clear ethical implications: patients and study volunteers should not be exposed to treatments that are based on skewed or otherwise inaccurate risk-benefit estimates [12].

For these and other reasons, it is now widely agreed that publication bias in favor of "statistically significant" findings poses a serious problem for academic research integrity [14-17]. In a recent attempt to estimate the extent of the prob-lem, researchers examined the fate of 221 studies from the social sciences that had been pre-registered in a database be-tween 2002 and 2012 [18]. They found that just 48% of the completed studies were ever published. To determine the rea-son for this disparity, the researchers contacted the authors of the study registrations. They asked whether their findings had ever been written up or submitted, and whether the obtained results were consistent with initial hypotheses.

Of all the studies with negative or null findings, only 20% were reported in a journal. Sixty-five percent had not been written up. By contrast, approximately 60% of the studies that provided support for initial hypotheses had been published. Many of the contacted authors said that they had not written up their findings because they thought journals would not publish them, or because the findings seemed "neither interesting nor important enough to warrant any further effort" [18].

These two explanations may be related. Often, the notion that negative findings are not "interesting or important enough" to be worth additional effort is grounded in a justified perception that most "top" journals would not publish such findings even if the researcher went to the trouble of writing them up. Evidently, the "prestige" issue mentioned above con-tinues to be a barrier for publishing negative results, with both journals (by failing to publish) and researchers (by failing to submit) contributing to a vicious cycle [19]. How might this cycle be broken?

One possibility is that all empirical research, not just clinical trials, conducted beyond the piloting stage should be pre-registered in a public repository. A requirement could then be imposed that a write-up-however brief-of the actual findings, whether positive or negative, must be appended to the registration when the data are available [20,21]. How to achieve such a system in practice is an open question. Howev-er, it would likely involve granting agencies, government of-fices, or universities and research institutes working in a "top-down" fashion to insist that all sponsored data, including data derived from animal studies, be published in some form.

A problem with this approach is that compliance would be difficult to ensure effectively. Already there is evidence that only 46% of a large subsample of trials on *ClinicalTri-als.gov*-the world's largest such repository-had reported results as of 2009 [22]. Moreover, in a systematic review looking at evidence from hundreds of studies covering several thousands of clinical trials dating back to the 1950s, researchers found that only about half of the trials had ever published results (with positive trials roughly twice as likely to be published as those yielding negative results) [23,24].

At the end of the day, top-down action by authoritative bodies to impose an obligation on all researchers would be a formidable undertaking. It could also lead to overly restrictive standards or expectations that scientists would feel pressured to conform to, even when doing so would lead to sub-optimal research practices [25-28]. These considerations do not entail that such an imposition should not be pursued in some form, but in the meantime, other options should also be considered. A possible "smaller-scale" approach would be to focus more at the level of individual journals, proposing policy alterations to encourage the submission and publication of negative results within their respective purviews.

In a recent paper, Locascio recommends a policy of "results blind evaluation" of manuscripts submitted to professional journals [29]. According to the proposed policy, reported results would be given no weight in the decision about whether the manuscript was suitable for publication. Instead, weight would be given exclusively to the judged importance of the research question and the quality of the study's methodology. Similar proposals have been advanced by others [30,31].

As a practical way of implementing such a policy, Locascio recommends a two stage process. In the first stage, the han-dling editor distributes just the Introduction and Methods sec-tions of a submitted manuscript to appropriate peer reviewers. A provisional decision about whether to accept or reject the manuscript is made on the basis of the initial reviews. In the second stage, the full manuscript is sent out, either to the same or different reviewers, "but only if the decision of the first stage is for acceptance with no more than minor revisions."

Such a policy, if it were widely adopted by journals, might indeed reduce bias against reports of null findings (but see [32]). However, a two-stage review process may seem too on-erous to implement for many journal editorial boards. Moreo-ver, it may increase the burden on unpaid peer reviewers, and would further elongate a review process that many authors find unacceptably slow already. It is therefore unclear whether such a policy will in fact be widely adopted.

Here, then, is an even more modest proposal-one that could be adopted by journals that decide not to embrace re-sults-blind publishing, or while transitioning to such a system. The proposal serves as a decisional heuristic for individual handling editors and peer reviewers, akin to the "reversal test" proposed Bostrom and Ord as a way of rooting out status quo bias in ethical reasoning [33]. It would require no additional time or resources for reviewers or editors and could be imple-mented tomorrow, without having to enact cumbersome changes to journal policies. We call it the *Publication Symmetry Test* (PST) and it is simply as follows:

Whenever editors or reviewers are proposing to accept a paper with a positive finding, they should ask themselves (ideally prompted as a forced question in the online review form) if they would be prepared to accept an identical paper with negative findings. Similarly, if proposing to reject a paper with negative findings, they should ask themselves if they would reject an identical paper with positive findings.

The idea is that a negative answer to either question raises the possibility of bias and should cause the editor or reviewer to reconsider their decision. For example, if an editor were unwilling to publish a negative version of the same study (for example because he or she judged it to be insufficiently interesting to readers), this may suggest that the editor is being unduly influenced by the perceived salience of positive findings. By contrast, if the editor is rejecting a paper with negative results (for example because he or she regards the statistical power as being too low), but would have been prepared to publish a positive version, this may imply that the editor is imposing too high a methodological standard on the negative publication.

It is important to recognize that identifying asymmetry in publication decisions is not necessarily a sign of bias. For ex-ample, it can be more difficult to prove a negative than a posi-tive, and some asymmetrical judgements may be due to this factor. To illustrate, there are some circumstances in which an intervention has a large effect size, and a study using a small sample can indicate an important positive result, while the same sample size could not exclude a clinically meaningful negative result. Nevertheless, a positive answer to the PST could serve as a potential trigger to re-evaluate the decision (or attempt to rule out any genuine asymmetries).

The PST would not eliminate publication bias. But it would help to raise awareness of it in a way that would neither put a heavy burden on journals to amend their processes of peer review, nor require top-down authorities to impose a sys-tem-wide constraint (i.e., pre-registration with enforced publi-cation of findings). We do not suggest that the latter strategies should not be pursued. But so long as the debates about their advisability and feasibility continue, more modest attempts at improving upon current practices are likely to be worth enacting [34,35]. This is especially the case for attempts that are easy to implement and have a very low risk of causing unexpected problems. The PST, we believe, fits this description.

## References

[1] Dickersin K (1990). The existence of publication bias and risk factors for its occurrence.. JAMA..

[2] Easterbrook PJ, Gopalan R, Berlin JA, Matthews DR (1991). Publication bias in clinical research.. The Lancet..

[3] Francis G (2013). Replication, statistical consistency, and publication bias.. J Math Psychol..

[4] Anderson G (2012). Why publish your negative results?.. On Medicine..

[5] Earp BD, Trafimow D (2015). Replication, falsification, and the crisis of confidence in social psychology.. Front Psychol..

[6] Earp BD, Everett JAC, Madva EN, Hamlin JK (2014). Out, damned spot:Can the "Macbeth Effect" be replicated?. Basic Appl Soc Psychol..

[7] Trafimow D (2014). Editorial.. Basic Appl Soc Psychol..

[8] Mahoney MJ (1977). Publication prejudices: an experimental study of confirmatory bias in the peer review system.. Cogn Ther Res..

[9] Firestein S (2015). Failure: Why Science Is So Successful.. Oxford: Oxford University Press.

[10] Heger M (2015). Editor's inaugural issue foreword: perspectives on translational and clinical research.. J Clin Transl Res..

[11] Earp JR (1927). The need for reporting negative results.. JAMA.

[12] Earp BD (2017). The need for reporting negative results - a 90 year update.. J Clin Transl Res..

[13] Kepes S, Banks GC, Oh I-S (2014). Avoiding bias in publication bias research: the value of "null" findings.. J Bus Psychol..

[14] Ioannidis JPA (2005). Why most published research findings are false.. PLoS Med..

[15] Greenwald AG (1975). Consequences of prejudice against the null hypothesis.. Psychol Bull..

[16] Ioannidis JPA (2006). Journals should publish all "null" results and should sparingly publish "positive" results.. Cancer Epidemiol Prev Biomark..

[17] Rosenthal R (1979). The file drawer problem and tolerance for null results.. Psychol Bull..

[18] Franco A, Malhotra N, Simonovits G (2014). Publication bias in the social sciences: unlocking the file drawer.. Science..

[19] Starbuck WH (2005). How much better are the most-prestigious journals? The statistics of academic publication.. Organ Sci..

[20] Chambers C, Munafo M (2013). Trust in science would be improved by study pre-registration.. The Guardian..

[21] Lash TL, Vandenbroucke JP (2012). Should preregistration of epidemio-logic study protocols become compulsory? Reflections and a counterproposal.. Epidemiology..

[22] Ross JS, Mulvey GK, Hines EM, Nissen SE, Krumholz HM (2009). Trial publication after registration in ClinicalTrials.gov: a cross-sectional analysis.. PLOS Med..

[23] Song F, Parekh S, Hooper L, Loke YK, Ryder J, Sutton AJ, Hing C, Kwok CS, Pang C, Harvey I (2010). Dissemination and publication of research findings: an updated review of related biases.. Health Technol Assess..

[24] (2015). AllTrials.. Half of all clinical trials have never reported results AllTrials..

[25] Alvarez RM (2014). The pros and cons of research preregistration.. OUPblog..

[26] Lash TL (2010). Preregistration of study protocols is unlikely to improve the yield from our science, but other strategies might.. Epidemiology..

[27] Scott S (2013). Pre-registration would put science in chains.. Times Higher Education..

[28] Trafimow D, Earp BD (2017). Null hypothesis significance testing and Type I error: the domain problem.. New Ideas Psychol..

[29] Locascio J Results blind science publishing.. Basic Appl Soc Psychol. In press;.

[30] Hanson R (2007). Conclusion-blind review.. Overcoming Bias..

[31] Findley MG, Jensen NM, Malesky EJ, Pepinsky TB (2016). Can results-free review reduce publication bias? The results and implications of a pilot study.. Comp Polit Stud..

[32] Teixeira da Silva JA (2016). Does the removal of results from a submitted paper reduce publication bias?. Pac Sci Rev B Humanit Soc Sci..

[33] Bostrom N, Ord T (2006). The reversal test: eliminating status quo bias in applied ethics.. Ethics..

[34] Everett JAC, Earp BD (2015). A tragedy of the (academic) commons: interpreting the replication crisis in psychology as a social dilemma for early-career researchers.. Front Psychol..

[35] LeBel EP, Vanpaemel W, McCarthy RJ, Earp BD, Elson M (2017). A unified framework to quantify the trustworthiness of empirical research.. PsyArXiv..

